# Male Circumcision and HIV in Africa

**DOI:** 10.1371/journal.pmed.0030074

**Published:** 2006-01-31

**Authors:** Taiwo Lawoyin, O. A Kehinde

**Affiliations:** **1**University College Hospital IbadanIbadanNigeria

We wish to congratulate Auvert et al. [ 1] on their work and wish them more success in their endeavors. Quite a number of studies have shown that circumcised males in heterosexual unions do have lower HIV rates [ 2–4]. It would be good to know to what extent circumcision affects the HIV rates in sub-Saharan African countries presently bearing the brunt of the disease.

Though ecological studies should be interpreted with caution, it would also be interesting to find out how this information helps us to better understand why some African countries with similar behavior have a much lower HIV rate than others [ 4–6]. West African countries, for example, have significantly higher circumcision rates than countries in the eastern and southern parts of Africa. HIV rates also appear to be generally lower in West Africa [ 7].

Also it would be interesting to find out if more African males who are not circumcised are ready to have this procedure done as a form of added protection, as there seem to be pockets of resistance to the procedure [ 8, 9].
